# Negative Prognostic Impact of Mineralocorticoid Receptor Antagonist in Elderly Patients Receiving TAVR

**DOI:** 10.3390/jcm12113742

**Published:** 2023-05-29

**Authors:** Teruhiko Imamura, Nikhil Narang, Hiroshi Onoda, Shuhei Tanaka, Ryuichi Ushijima, Mitsuo Sobajima, Nobuyuki Fukuda, Hiroshi Ueno, Koichiro Kinugawa

**Affiliations:** 1 The Second Department of Internal Medicine, University of Toyama, Toyama 930-0194, Japan; 2 Advocate Christ Medical Center, Oak Lawn, IL 60453, USA

**Keywords:** heart failure, hemodynamics, congestion, aortic valve disease

## Abstract

**Background:** Morbidity and mortality following trans-catheter aortic valve replacement (TAVR) remain high. Renin-angiotensin system inhibitors improve clinical outcomes in the cohort studied in this work. However, post-TAVR prognostic impact of mineralocorticoid receptor antagonist (MRA), another neuro-hormonal blocker, remains uncertain. Here, we hypothesized that MRA was associated with improved clinical outcomes in elderly patients with severe aortic stenosis receiving TAVR. Methods: Consecutive patients who received TAVR at our institute between 2015 and 2022 were considered for inclusion. Propensity score matching analysis was performed to match pre-procedural baseline characteristics between those with and without MRA. The prognostic impact of MRA use on the composite primary endpoint consisting of all-cause death and heart failure during the 2-year observational period following index discharge was evaluated. Results: Among 352 patients who received TAVR, 112 patients (median 86 years, 31 men) were included, consisting of baseline-matched 56 patients with MRA and 56 patients without MRA. Following TAVR, patients with MRA had more impaired renal function compared with no MRA group. Following index discharge, serum potassium tended to increase, and renal function tended to decline in patients with MRA. Patients with MRA had a higher cumulative incidence of the primary endpoints during a two-year observational period (30% versus 8%, *p* = 0.022). Conclusions: Routine prescription of MRA might not be recommended in elderly patients with severe aortic stenosis receiving TAVR, given its negative prognostic impact. Optimal patient selection for MRA administration in this cohort needs further study.

## 1. Background

Given the recent innovation of trans-catheter aortic valve replacement (TAVR) together with sophisticated pre-procedural imaging modalities including contrast computed tomography [1], symptomatic severe aortic stenosis can be treated in higher-risk elderly patients with multiple comorbidities including chronic lung and kidney disease [2,3]. The indication of TAVR has expanded from initial indication of high-surgical risk cohorts to intermediate and low-surgical risk cohorts after subsequent data suggesting non-inferiority of TAVR to surgical aortic valve replacement [4]. Nevertheless, the incidence of readmission following TAVR due to worsening heart failure still occurs at a non-negligible rate [5]. Anatomical intervention to a severely stenotic aortic valve is just part of the management strategy needed for patients with left ventricular dysfunction at the time of TAVR—optimization of heart failure-specific therapies is additionally needed, which, if done adequately, can improve post-TAVR clinical outcomes [6].

Renin-angiotensin system (RAS) inhibitors—including angiotensin-converting enzyme II inhibitor, angiotensin receptor blocker, and the recently-introduced angiotensin receptor neprilysin inhibitor—are essential therapies for the management of patients with heart failure with reduced ejection fraction (HFrEF) [7]. It is worth noting that angiotensin receptor neprilysin inhibitor was found to improve clinical outcomes in patients with a wide range of left ventricular ejection fraction (LVEF) [8]. Recently, RAS inhibitors were demonstrated to improve mortality following TAVR in several studies [6,9,10,11].

Mineralocorticoid receptor antagonist (MRA) is another established therapy that should reduce cardiovascular mortality and risk of heart failure hospitalizations for those with HFrEF [12,13]. Although evidence is less robust [14], MRA is often administered in patients with heart failure with preserved ejection fraction (HFpEF), given heterogeneity of treatment effect seen in different clinical trial sites. Given the pharmacological mechanism that MRA also suppresses the RAS cascade and facilitates cardiac reverse remodeling [15], we hypothesized that MRA might also improve clinical outcomes following TAVR. In this study, we used prospectively constructed registry data to evaluate the prognostic impact of MRA therapy in patients receiving TAVR versus background-matched cohorts receiving TAVR without MRA.

## 2. Materials and Methods

### 2.1. Patient Selection

Patients who underwent TAVR at our institute between 2015 and 2022 were included in our prospective institutional registry database. This study used this prospectively collected data and was conducted retrospectively. Patients who died during index hospitalization were excluded given the lack of their follow-up. Patients who lost follow-up were also excluded. Written informed consents were obtained from all participants on admission before being included in our registry database. The institutional review board approved the study protocol. 

### 2.2. TAVR Procedure

Patients with symptomatic severe aortic stenosis with aortic valve peak velocity >4.0 m/s and mean aortic valve velocity >40 m/sec were considered to receive TAVR. The indication for TAVR was determined based on the consensus of a multi-disciplinary team consisting of cardiac surgeons, interventional cardiologists, anaesthesiologists, and imaging specialists. Patients received TAVR using Edward Sapien XT/Sapien 3 Transcatheter Heart Valve (Edwards Lifesciences, Irvine, CA, USA) or Medtronic CoreValve/Evolut R Revolving System (Medtronic, Minneapolis, MN, USA) via trans-femoral approach or alternative approach under the general or local anesthesia. Following the procedure, all patients received guideline-directed standard medical therapy. 

### 2.3. Propensity Score Matching

The independent variable in this study was defined as the use of MRA at index discharge. The indication of MRA use was heart failure and was at the discretion of the attending cardiologists. Briefly, MRA is contraindicated for those with hyperkalemia, hypotension, or severe renal disease. We performed a propensity score analysis, matching for age, baseline estimated glomerular filtration rate, and baseline left ventricular ejection fraction to collect 1:1 of the MRA group, who used MRA at index discharge, and the no MRA group, who did not use MRA at index discharge, given the impact of these variables on the indication of MRA use. A propensity score was calculated using logistic regression modeling, including all three of the above variables, and paired participants with a propensity score within 0.20 in each group were selected. 

### 2.4. Data Collection

All clinical data were retrieved from the institutional registry database. Demographics, comorbidities, echocardiography, and laboratory data obtained before TAVR procedures were collected as baseline characteristics. Data following TAVR, including laboratory, echocardiography, and medication variables, were also collected. Of note, the use of MRA following TAVR procedures was defined as an independent variable. 

The primary outcome was defined as a composite of all-cause death and heart failure readmissions that required IV diuretic therapy following index discharge (defined as day 0). As secondary outcomes, trends in serum potassium, estimated glomerular filtration rate, and plasma B-type natriuretic peptide level at index discharge, 3 months later, and 6 months later were defined. 

### 2.5. Statistical Analysis

Continuous variables were presented as median (IQR) and compared using Mann–Whitney U test. Thus, all continuous variables were assumed as non-parametric irrespective of their distributions given the small sample size. Categorical variables were presented as number of cases (percentage of the total) and compared using Fisher’s exact test. A value of 2-tailed *p* < 0.05 was considered statistically significant. Statistical analyses were performed using SPSS Statistics 22 (SPSS Inc., Armonk, IL, USA) and EZR, which is a modified version of R commander designed to add statistical functions frequently used in biostatistics.

The independent variable was defined as MRA use at index discharge. The primary endpoint was defined as a composite of all-cause death and heart failure readmissions following index discharge (day 0). Propensity score matching analysis was performed as detailed above to match pre-procedural baseline characteristics between those with and without MRA. 

Log-rank analysis was performed to compare the cumulative incidence of the primary endpoint between those with and without MRA. Cox proportional hazard ratio regression analysis was performed to investigate the impact of MRA use on the primary endpoint. Variables that were considered to be potentially associated with clinical outcomes were included in the univariable analyses. Variables with *p* < 0.05 in the univariable analyses were included in the multivariable analysis. Sub-analyses were performed to calculate the hazard ratio in each sub-groups. Sub-groups and their cutoffs were pre-specified considering the impact on MRA: estimated glomerular filtration ratio, left ventricular ejection fraction, systolic blood pressure, use of RAS inhibitor, and heart failure history. Their results were displayed on the forest plot and interactions between the groups divided by the cutoffs were analyzed and stated. Trends of clinical variables following index discharge were assessed using Friedman test.

## 3. Results

### 3.1. Baseline Characteristics

A total of 352 patients who received TAVR at our institute were considered for inclusion. Patients who died during the index hospitalization and those without follow-up data were excluded. Following propensity score matching analysis to match clinically important pre-procedural baseline characteristics, 112 patients were statistically selected, consisting of 56 patients with MRA and 56 without MRA (Table 1).

The median age for the cohort was 86 (84, 89) years old; 31 people in the cohort (28%) were men. The median STS score was 5.0 (4.0, 6.5). The median estimated glomerular filtration rate was 47 (35, 58) mL/min/1.73 m^2^. The median aortic valve peak velocity was 4.5 (4.1, 4.9) m/sec. Most patients had preserved left ventricular ejection fraction with a median value of 63% (55%, 70%). There were no statistically significant differences in pre-procedural baseline characteristics, except for lower systolic blood pressure in patients with MRA (*p* = 0.003). 

### 3.2. Post-Procedural Data

Following TAVR, 56 patients received MRA and the other 56 patients did not receive MRA (Table 2). Median aortic valve peak velocity was 2.0 (1.7, 2.3) m/s. Patients with MRA had a lower estimated glomerular filtration rate compared with those without MRA (*p* = 0.048). More patients with MRA received loop diuretics compared with those without MRA (*p* < 0.001).

### 3.3. Primary Outcomes

**Figure 1:**
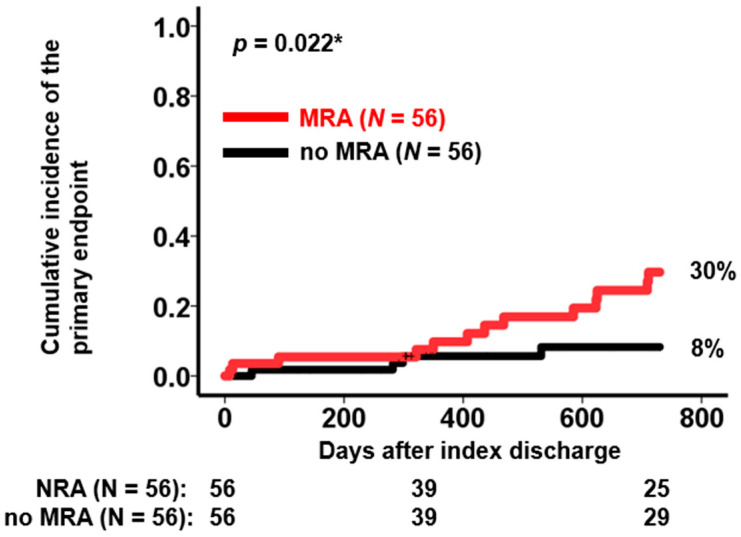
Cumulative incidence of the primary endpoint stratified by the prescription of MRA at index discharge. MRA, mineralocorticoid receptor antagonist. Patients with MRA had a significantly higher cumulative incidence of the primary endpoint following index discharge. The primary endpoint was defined as all-cause death or heart failure readmissions. * *p* < 0.05 by log-rank test.

During a 2-year observational period (median duration 730 (356, 730) days) following index discharge, 17 patients experienced the primary endpoint (8 deceased patients, 7 patients with heart failure readmissions, and 2 patients who had heart failure readmissions and died later). A cumulative incidence of the primary endpoint was significantly higher in patients with MRA compared with those without MRA during a 2-year observational period (30% versus 8%, *p* = 0.022; Figure 1). Among potential variables, MRA use was associated with the primary endpoint with a hazard ratio of 3.42 (95% confidence interval 1.11–10.5, *p* = 0.032) in addition to estimated glomerular filtration rate and loop diuretics use in the univariable analysis (Table 3). However, all of these variables did not reach statistical significance to predict the primary endpoint in the multivariable analysis (*p* > 0.05 for all).

### 3.4. Sub-Analyses for the Primary Endpoint

**Figure 2:**
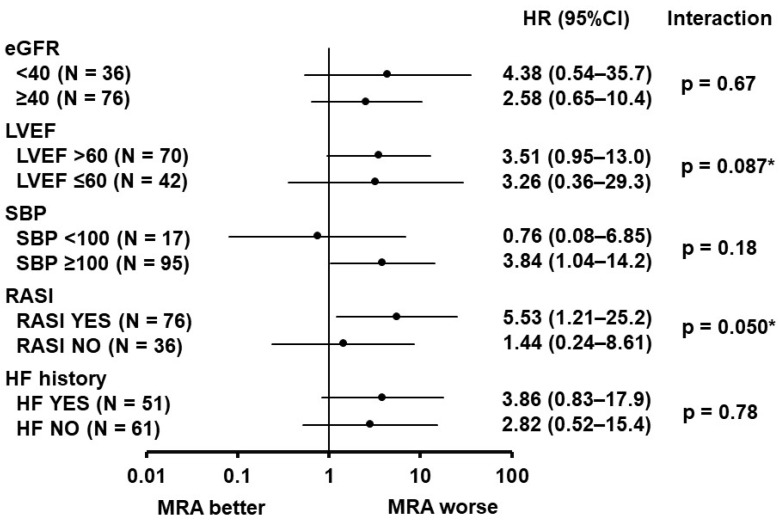
Prognostic impact of MRA use on the primary endpoint in each sub-group. HR, hazard ratio; CI, confidence interval; eGFR, estimated glomerular filtration rate; LVEF, left ventricular ejection fraction; SBP, systolic blood pressure; RASI, renin-angiotensin system inhibitor; HF, heart failure. Clinical variables that were associated with the indication of MRA administration obtained on admission were included to stratify patients’ cohorts. * *p* < 0.10 for interaction between the two groups.

A hazard ratio of MRA use to predict the incidence of the primary endpoint was displayed in each sub-group stratified by clinically important variables (Figure 2). There were no significant interactions between the sub-groups. In other words, negative prognostic impacts of MRA use were observed in a variety of clinical sub-groups. Nevertheless, the hazard ratio tended to be higher in patients with RAS inhibitors compared with those without RAS inhibitors (5.53 (95% confidence interval 1.21–25.2) versus 1.44 (95% confidence interval 0.24–8.61), interaction *p* = 0.050).

### 3.5. Trends in Secondary Outcomes

**Figure 3:**
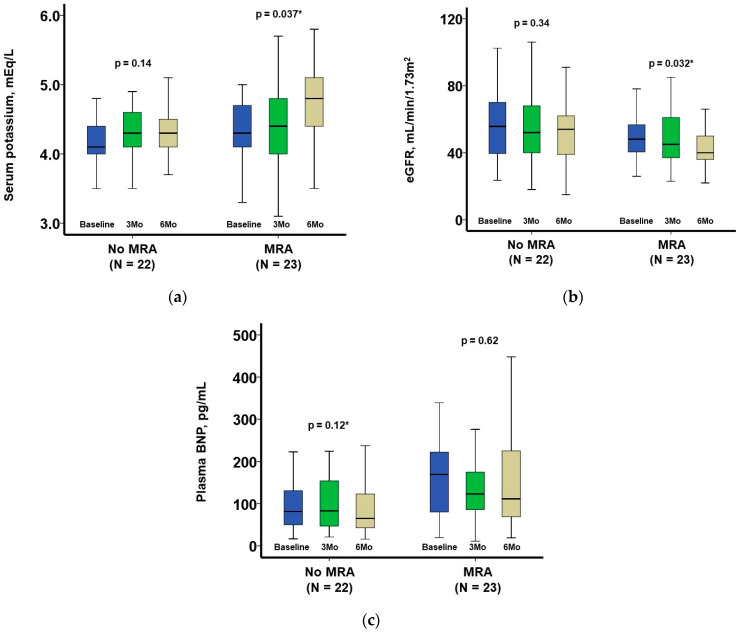
The trend in clinical variables following index discharge among those with and without MRA: serum potassium (**a**), eGFR, (**b**), and plasma BNP (**c**). MRA, mineralocorticoid receptor antagonist; eGFR, estimated glomerular filtration rate; BNP, B-type natriuretic peptide. Only patients with complete follow-up data (baseline, 3 months, and 6 months later) were included in this analysis to assess data trends. Serum potassium levels increased and glomerular filtration rate decreased in patients receiving MRA, whereas these variables remained unchanged in patients without MRA. Plasma B-type natriuretic peptide level remained unchanged in patients receiving MRA, whereas it improved in patients without MRA. * *p* < 0.05 by Friedman test for data trend.

Serum potassium levels increased in patients with MRA, whereas they remained unchanged in patients without MRA (Figure 3A). The estimated glomerular filtration rate decreased during 6-month MRA therapy, whereas it remained unchanged in patients without MRA (Figure 3B). Plasma B-type natriuretic peptide remained unchanged in patients with MRA, whereas it decreased significantly in patients without MRA (Figure 3C).

## 4. Discussion

In this retrospective study using prospectively collected registry data, we investigated the prognostic impact of MRA use following TAVR in elderly patients with severe aortic stenosis. Paradoxically, the MRA therapy was associated with a higher incidence of the primary composite endpoint consisting of all-cause death and heart failure readmissions during the two-year observational period following post-TAVR index discharge, as compared with baseline-matched cohorts without MRA also receiving TAVR. MRA was associated with worse clinical outcomes particularly when administered concomitantly with RAS inhibitors. MRA therapy was associated with incremental serum potassium level and gradual decline in renal function following post-TAVR index discharge. 

### 4.1. Neuro-Hormonal Blockade in Patients Receiving TAVR

Neuro-hormonal blockers, including RAS inhibitors and beta-blockers, are established pharmacological therapy for those with HFrEF, based on the concept that neuro-hormonal systems are inappropriately activated in chronic heart failure leading to worsening pulmonary congestion [7]. The concept is expanded to HFpEF and even with angiotensin receptor neprilysin inhibitors which may improve outcomes specifically in patients with LVEF < 60% and in females based on clinical trial data [8].

The RAS signal cascade is activated in patients with aortic stenosis and even those following TAVR, and RAS inhibitors theoretically would be effective in this cohort [16,17]. In a large cohort of patients with severe symptomatic aortic stenosis from the PARTNER 2 trial, RAS inhibitor treatment at baseline was independently associated with a lower incidence of 2-year all-cause and cardiovascular mortality [9]. One of the mechanisms of this finding is explained by greater left ventricular mass regression by continued RAS blockade therapy following TAVR [11]. The beneficial impact of post-TAVR RAS inhibitors on improving survival is dose-dependent [10]. On the contrary, beta-blockers, another neuro-hormonal blocker, did not add an incremental reduction in the two-year all-cause mortality upon RAS inhibitor in patients receiving TAVR [6]. As discussed in the literature, the negative chronotropic effect of beta-blockers in patients with diastolic dysfunction may lead to a decrease in cardiac output and worsen functional status and clinical outcomes [18].

### 4.2. Prognostic Impact of MRA following TAVR

With the favorable impact of RAS inhibitors in the TAVR candidates, MRA was observed to have a negative prognostic impact following TAVR in this study. The TOPCAT trial demonstrated that MRA reduced the incidence of heart failure hospitalization in patients with HFpEF, although it could not demonstrate the impact of MRA on the primary composite endpoint [14]. The physiology of post-TAVR patients is not similar to those of HFpEF. Moreover, the participants of this trial were younger (68 years versus 86 years) and had more preserved renal function (glomerular filtration rate 65 versus 47 mL/min/1.73 m^2^) compared with the patients in this cohort, which consisted of standard elderly patients with severe aortic stenosis with multiple comorbidities. 

The safety and efficacy of MRA in elderly patients remain controversial. A meta-analysis demonstrated that the impact of MRA on mortality in patients with HFrEF aged ≥75 years was uncertain [19]. There was no observed benefit with MRA treatment in patients with HFpEF aged ≥65 years. On the contrary, a recent study demonstrated that additional MRA therapy was associated with lower all-cause mortality in patients with HFrEF aged ≥80 years [20]. Of note, hyperkalemia was often observed in elderly patients receiving MRA. Consistently, hyperkalemia and renal impairment further progressed during MRA therapy in our cohort. These unfavorable adverse events may occur more often in elderly patients receiving MRA. 

In our study, sub-analyses demonstrated that concomitant use of RAS inhibitors tended to be more harmful. Adverse events, including hyperkalemia and renal impairment, might be enhanced when RAS inhibitors and MRA are concomitantly administered in elderly patients [21]. Hyperkalemia and renal impairment may have directly had worse prognostic effect, and/or the doses of concomitant RAS inhibitors may have been unintentionally down-titrated. Although we could not find obvious inter-group differences in the impact of MRA, further studies are warranted to clarify sub-groups that may benefit from MRA among those receiving TAVR. 

### 4.3. Limitations

This is a retrospective study consisting of a small sample size, which might have affected the results of several analyses. Several non-significant findings in this study may turn significant in the larger-scale studies. We attempted to match baseline characteristics between the two groups, but other uninvestigated confounders might have affected the findings of this study. Of note, several baseline characteristics tended to be worse in patients with MRA compared with those without MRA. Thus, prospective randomized control trials are warranted to demonstrate the prognostic impact of MRA following TAVR. We highly recommend strictly selecting candidates of MRA therapy among the current TAVR candidates. We defined the use of MRA at index discharge as an independent variable. We did not consider the type and dose of MRA. We did not consider dose adjustment and termination/initiation of MRA during the observational period following index discharge. Although not indicated for heart failure, several MRAs, including esaxerenone and finerenone, are recently available. The prognostic impact of these MRAs for those receiving TAVR remains unknown.

## 5. Conclusions

MRA treatment was associated with incremental mortality and heart failure recurrence following TAVR in elderly patients with severe aortic stenosis, possibly due to MRA-related hyperkalemia and renal impairment. Routine MRA treatment for all TAVR candidates might not be recommended. Further studies are warranted to find the optimal patient selection and adjustment of MRA therapy in this cohort.

## Figures and Tables

**Table 1:** Preoperative baseline characteristics.

	Total (*N* = 112)	MRA (*N* = 56)	No MRA (*N* = 56)	*p* Value
Demographics				
Age, ears	86 (84, 89)	86 (84, 89)	86 (84, 89)	1.0
Men	31 (28%)	18 (32%)	13 (23%)	0.20
Body surface area, m^2^	1.38 (1.25, 1.47)	1.39 (1.29, 1.53)	1.35 (1.24, 1.43)	0.076
Systolic blood pressure, mmHg	117 (106, 130)	115 (100, 121)	120 (112, 131)	0.003 *
Pulse rate, bpm	71 (63, 77)	68 (61, 75)	72 (64, 79)	0.086
STS score	5.0 (4.0, 6.5)	5.5 (4.5, 7.3)	4.1 (3.9, 5.4)	0.064
Comorbidity				
Hypertension	82 (73%)	44 (79%)	38 (68%)	0.14
Dyslipidemia	55 (49%)	24 (43%)	31 (55%)	0.13
Diabetes mellitus	23 (21%)	13 (23%)	10 (18%)	0.32
Coronary artery disease	26 (23%)	12 (21%)	14 (25%)	0.41
History of stroke	19 (17%)	9 (16%)	10 (18%)	0.50
History of heart failure	51 (46%)	29 (52%)	22 (39%)	0.13
Atrial fibrillation	19 (17%)	13 (23%)	6 (11%)	0.076
Peripheral artery disease	29 (26%)	17 (30%)	12 (21%)	0.19
Laboratory data				
Hemoglobin, g/dL	11.4 (10.2, 12.4)	11.6 (10.4, 12.6)	10.9 (10.1, 12.2)	0.17
Serum albumin, g/dL	3.8 (3.5, 4.0)	3.8 (3.6, 4.0)	3.8 (3.5, 3.9)	0.27
Serum sodium, mEq/L	140 (139, 142)	140 (138, 142)	140 (139, 142)	0.49
Serum potassium, mEq/L	4.4 (4.1, 4.6)	4.4 (4.1, 4.6)	4.4 (4.2, 4.7)	0.54
eGFR, mL/min/1.73 m^2^	47 (35, 58)	47 (35, 59)	50 (36, 63)	0.36
Plasma BNP, log pg/mL	2.4 (2.1, 2.7)	2.5 (2.2, 2.7)	2.3 (2.1, 2.7)	0.081
Echocardiography				
LVDd, mm	45 (42, 50)	46 (43, 53)	45 (42, 47)	0.15
LVEF, %	63 (55, 70)	63 (53, 70)	65 (55, 70)	0.41
Aortic valve peak velocity, m/s	4.5 (4.1, 4.9)	4.4 (4.0, 4.9)	4.6 (4.2, 4.9)	0.68

MRA, mineralocorticoid receptor antagonist; STS, society for thoracic surgeons; eGFR, estimated glomerular filtration rate; BNP, B-type natriuretic peptide; LVDd, left ventricular end-diastolic diameter; LVEF, left ventricular ejection fraction. Continuous variables are displayed as median and interquartile and compared between the two groups using Mann-Whitney U test. Categorical variables are displayed as numbers and percentages and compared between the two groups using Fischer’s exact test. * *p* < 0.05.

**Table 2:** Post-procedural data.

	Total (*N* = 112)	MRA (*N* = 56)	No MRA (*N* = 56)	*p* Value
Laboratory data				
Hemoglobin, g/dL	10.3 (9.6, 11.3)	10.4 (9.7, 11.4)	10.3 (9.4, 11.1)	0.56
Serum albumin, g/dL	3.3 (3.1, 3.6)	3.4 (3.2, 3.7)	3.2 (3.1, 3.5)	0.058
Serum sodium, mEq/L	139 (137, 141)	139 (136, 140)	140 (138, 141)	0.054
Serum potassium, mEq/L	4.3 (4.0, 4.6)	4.3 (4.0, 4.7)	4.2 (4.0, 4.4)	0.26
eGFR, mL/min/1.73 m^2^	49 (37, 60)	44 (36, 58)	53 (38, 65)	0.048 *
Plasma BNP, log pg/mL	2.1 (1.8, 2.4)	2.2 (1.9, 2.5)	2.0 (1.7, 2.3)	0.068
Echocardiography				
LVDd, mm	45 (41, 48)	44 (41, 49)	45 (41, 48)	0.71
LVEF, %	65 (59, 71)	65 (57, 71)	66 (60, 72)	0.90
Aortic valve peak velocity, m/s	2.0 (1.7, 2.3)	2.0 (1.7, 2.3)	2.0 (1.6, 2.3)	0.75
Medications				
Beta-blocker	42 (38%)	25 (45%)	17 (30%)	0.12
RAS inhibitor	76 (68%)	38 (68%)	38 (68%)	1.0
MRA	56 (50%)	56 (100%)	0 (0%)	-
Loop diuretics	56 (50%)	39 (70%)	17 (30%)	<0.001 *
Thiazide	3 (3%)	1 (2%)	2 (4%)	0.56
Statin	60 (54%)	27 (48%)	33 (59%)	0.17

MRA, mineralocorticoid receptor antagonist; eGFR, estimated glomerular filtration rate; BNP, B-type natriuretic peptide; LVDd, left ventricular end-diastolic diameter; LVEF, left ventricular ejection fraction. RAS, renin-angiotensin system. Continuous variables are displayed as median and interquartile and compared between the two groups using Mann-Whitney U test. Categorical variables are displayed as numbers and percentages and compared between the two groups using Fischer’s exact test. * *p* < 0.05.

**Table 3:** Prognostic impact of post-TAVR clinical variables on the primary outcome.

	Univariable Analyses		Multivariable Analyses	
	Hazard Ratio (95% CI)	*p* Value	Hazard Ratio (95% CI)	*p* Value
Age, years	1.04 (0.91–1.19)	0.57		
History of heart failure	1.80 (0.66–4.87)	0.25		
eGFR, mL/min/1.73 m^2^	0.97 (0.94–0.99)	0.041 *	0.98 (0.95–1.01)	0.23
Plasma BNP, log pg/mL	3.09 (0.81–11.8)	0.099		
LVEF, %	1.01 (0.96–1.06)	0.83		
RAS inhibitor use	1.18 (0.42–3.35)	0.76		
Loop diuretics use	3.71 (1.21–11.4)	0.022 *	2.17 (0.64–7.40)	0.21
MRA use	3.42 (1.11–10.5)	0.032 *	2.20 (0.67–7.30)	0.20

CI, confidence interval; eGFR, estimated glomerular filtration rate; BNP, B-type natriuretic peptide; LVEF, left ventricular ejection fraction; MRA, mineralocorticoid receptor antagonist. Variables obtained at index discharge following TAVR, which were considered to have potential prognostic impact, were included. Variables with *p* < 0.05 in the univariable analyses were included in the multivariable analysis. * *p* < 0.05 by Cox proportional hazard ratio regression analysis.

## Data Availability

Data are available upon reasonable request from the corresponding author.
